# Supplementary material for: Activation of individual L1 retrotransposon instances is restricted to cell-type dependent permissive loci
Source: eLife. 2016 Mar 26;5:e13926. doi: 10.7554/eLife.13926 (PMC4866827; doi:10.7554/eLife.13926)
Supplement: Supplementary file 1. — Note that HEK-293T data were ambiguously named in the original publication, as 'HEK-293' in the main text, but as 'HEK-293T' in the method section (Sultan et al., 2014). We solved this ambiguity by searching for RNA-seq reads matching the SV40 virus and Neomycin-resistance gene sequences, which confirmed the nature of the cells as being 'HEK-293T'. DOI: http://dx.doi.org/10.7554/eLife.13926.016 [file elife-13926-supp1.zip › Supplementary_file_1.pdf]

Name	Description	Origin	Growth medium base	RNA-seq data		
				Database	Accession Number	Reference
HeLa S3	Human cervix carcinoma (clonal derivative of HeLa cells selected to grow in suspension)	ECACC (distributed by Sigma)	DMEM	ENCODE	ENCSR000CPR	ENCODE Project
MCF7	Human breast adenocarcinoma	ECACC (distributed by Sigma)	DMEM	ArrayExpress, ENCODE	E-MTAB-3788, ENCSR000CPT	This study, ENCODE Project
Hep G2	Human hepatocarcinoma	ECACC (distributed by Sigma)	DMEM	ENCODE	ENCSR000CPE	ENCODE Project
HEK-293	Human embryonic kidney	ECACC (distributed by Sigma)	DMEM	n/a	n/a	n/a
MRC-5	Human lung primary fibroblasts	ECACC (distributed by Sigma)	DMEM	n/a	n/a	n/a
2102Ep	Human embryonal carcinoma	Kind gift of P. W. Andrews (University of Sheffield, UK)	DMEM	ArrayExpress	E-MTAB-3788	This study
IMR-90	Lung primary fibroblasts	ATCC (distributed by LGC Standards)	DMEM	ENCODE	ENCSR000CTQ	ENCODE Project
BJ	Human forskin primary fibroblasts	ATCC (distributed by LGC Standards)	DMEM	ENCODE	ENCSR000COP	ENCODE Project
HEK-293T	Human embryonic kidney also known as 293tsA1609neo clonal derivative of HEK-293 cells transformed with the SV40 Large T-antigen	ATCC (kind gift of Andrea Cimarelli, ENS-Lyon, France)	DMEM	ENA	PRJEB4197	Sultan M et al. BMC Genomics (2014)
HCT 116	Human colon carcinoma	ECACC (distributed by Public Health England)	McCoy's 5A (modified)	ENA	PRJNA252833	Sanchez Y et al. Nat Commun (2014)
K-562	Human chronic myelogenous leukaemia	ECACC (distributed by Public Health England)	RPMI 1640 (ATCC modification)	ENCODE	ENCSR000CPH	ENCODE Project
H1 hESC	Human embryonic stem cells	Cells not available for ethical reasons. Genomic DNA was a kind gift of J.L. Garcia Perez (Genyo, University of Granada, Spain)	n/a	ENCODE	ENCSR000COU	ENCODE Project

Supplementary Table 1. Description of the cell lines and RNA-seq datasets used in this study.
